# The Effector Repertoire of the Hop Downy Mildew Pathogen *Pseudoperonospora humuli*

**DOI:** 10.3389/fgene.2020.00910

**Published:** 2020-08-11

**Authors:** Savithri Purayannur, Liliana M. Cano, Megan J. Bowman, Kevin L. Childs, David H. Gent, Lina M. Quesada-Ocampo

**Affiliations:** ^1^Department of Entomology and Plant Pathology, North Carolina State University, Raleigh, NC, United States; ^2^Indian River Research and Education Center, Department of Plant Pathology, Institute of Food and Agricultural Sciences, University of Florida, Fort Pierce, FL, United States; ^3^Department of Plant Biology, Michigan State University, East Lansing, MI, United States; ^4^Ball Horticultural Company, West Chicago, IL, United States; ^5^United States Department of Agriculture-Agricultural Research Service, Forage Seed and Cereal Research Unit, Oregon State University, Corvallis, OR, United States

**Keywords:** downy mildew, *Pseudoperonospora*, secretome, effectors, RXLR

## Abstract

*Pseudoperonospora humuli* is an obligate biotrophic oomycete that causes downy mildew (DM), one of the most destructive diseases of cultivated hop that can lead to 100% crop loss in susceptible cultivars. We used the published genome of *P. humuli* to predict the secretome and effectorome and analyze the transcriptome variation among diverse isolates and during infection of hop leaves. Mining the predicted coding genes of the sequenced isolate OR502AA of *P*. *humuli* revealed a secretome of 1,250 genes. We identified 296 RXLR and RXLR-like effector-encoding genes in the secretome. Among the predicted RXLRs, there were several WY-motif-containing effectors that lacked canonical RXLR domains. Transcriptome analysis of sporangia from 12 different isolates collected from various hop cultivars revealed 754 secreted proteins and 201 RXLR effectors that showed transcript evidence across all isolates with reads per kilobase million (RPKM) values > 0. RNA-seq analysis of OR502AA-infected hop leaf samples at different time points after infection revealed highly expressed effectors that may play a relevant role in pathogenicity. Quantitative RT-PCR analysis confirmed the differential expression of selected effectors. We identified a set of *P. humuli* core effectors that showed transcript evidence in all tested isolates and elevated expression during infection. These effectors are ideal candidates for functional analysis and effector-assisted breeding to develop DM resistant hop cultivars.

## Introduction

Downy mildew (DM) pathogens are a group of obligate biotrophic oomycetes that belong to the Peronosporales lineage oomycetes and have caused epidemics in many agriculturally important plants including grapes (Gessler et al., 2011), spinach (Correll et al., 2011), cucumber (Holmes et al., 2015), and lettuce (Parra et al., 2016), to name a few. Despite their economic importance, DM pathogens have been relatively under-examined because they cannot be cultured on artificial media and are difficult to transform. Most DM research has been conducted on the *Arabidopsis* DM pathogen *Hyaloperonospora arabidopsidis* due to the wealth of genetic information available on the model plant, *Arabidopsis* (Kamoun, 2006). Advancements in genome sequencing have facilitated development of genomic resources for several economically important DM pathogens in the past decade. These include *Pseudoperonospora cubensis* (cucurbit DM) (Savory et al., 2012a, b), *Peronospora tabacina* (tobacco DM) (Derevnina et al., 2015), *Plasmopara halstedii* (sunflower DM) (Sharma et al., 2015), *Plasmopara viticola* (grape DM) (Dussert et al., 2016, 2019), *Bremia lactucae* (lettuce DM) (Fletcher et al., 2019), and *Pseudoperonospora humuli* (hop DM) (Rahman et al., 2019).

During infection on host plants, oomycetes secrete molecules called effectors that alter host cell machinery to allow colonization (Bozkurt et al., 2012). In resistant hosts, effectors can be directly or indirectly recognized by *R* proteins, in which case an effector acts as an avirulence factor that activates effector-triggered immunity (ETI) in host plants (Jones and Dangl, 2006). This avirulence function of effectors can be used in plant improvement programs to search for genotypes that contain *R* genes against core *in planta* expressed pathogen effectors (Jones et al., 2014; Vleeshouwers and Oliver, 2014).

Based on host cell localization, oomycete effectors are classified into apoplastic and cytoplasmic effectors, those localizing to the plant apoplast and those translocated into the host cell, respectively (Schornack et al., 2009). Apoplastic effectors are enzymes and enzyme inhibitors that bind to host-secreted defense molecules (Sperschneider et al., 2017). Oomycete cytoplasmic effectors are modular proteins that are distinguished by conserved domains. The most well-studied class of cytoplasmic effectors are the RXLRs, which possess an Arg-X-Leu-Arg (RXLR) amino acid motif positioned within 40 amino acids downstream of the secretion signal followed by a Glu-Glu-Arg (EER) motif (Bozkurt et al., 2012). The RXLR motif bears a striking similarity to the *Plasmodium falciparum* PEXEL proteins that contain a characteristic RXLXE/D/Q motif (Hiller et al., 2012) and the *Toxoplasma gondii* TEXEL motif (RRLXX) (Coffey et al., 2015). RXLR effector proteins are enriched in the Peronosporales clade, which contains *Phytophthora* and the DM pathogens (Thines and Kamoun, 2010; Thines, 2014). Several evolutionary scenarios such as gene duplication, recombination events and point mutation scenarios that can promote diversification of RXLR effectors within Peronosporales have been described (Jiang et al., 2008; Goss et al., 2013). A level of degeneracy can occur in the RXLR motif as evidenced by the RVRN motif in *H*. *arabidopsidis* (Bailey et al., 2011), QXLRs in *P*. *cubensis* (Tian et al., 2011), GKLR in *B*. *lactucae* (Stassen et al., 2013), and RXLKs in *P. halstedii* (Sharma et al., 2015).

The function of the RXLR motif is highly debated (Ellis and Dodds, 2011). This motif is thought to enable host cell targeting in a mechanism yet to be described. The role of the RXLR motif in host cell translocation was proposed in the *Phytophthora infestans* effector Avr3a (Whisson et al., 2007) whereas in the *Phytophthora sojae* effector AVR1b, the RXLR motif was shown to mediate binding to phosphatidylinositol phosphates for host cell uptake (Kale et al., 2010). However, recent research suggests that the RXLR motif is cleaved before secretion in Avr3a and is involved in secretion from the pathogen rather than translocation into the host cell (Wawra et al., 2017).

To add another level of complexity to the RXLR effector domain features, the C-termini of some RXLRs have combinations of the hydrophobic amino acids W, Y, and L forming a WY domain (Jiang et al., 2008; Haas et al., 2009). This domain forms a conserved structural feature and may also be present in tandem repeats joined by linker sequences (Boutemy et al., 2011; Chou et al., 2011). The WY domain is thought to mediate the “effector” function of RXLRs and suppress host immune signaling by various mechanisms, like suppression of cell death in the *P*. *sojae* effector Avr1b (Dou et al., 2008), interaction with the E3 ligase CMPG1 in the *P*. *infestans* effectors Avr3a (Bos et al., 2010), and interaction with MAPKKK in the *P*. *infestans* PexRD2 (King et al., 2014). Mutations in the WY domain abolish the interaction of the Avr3a (Bos et al., 2010) and PexRD2 (King et al., 2014) effectors with their targets. A recent report shows the role of the WY domain in effector dimerization and subsequent virulence activity (Guo et al., 2019).

Recently, genome and effectorome analyses of DM pathogens revealed the presence of WY domain-containing effectors that lacked a canonical RXLR motif in *P. tabacina* (Derevnina et al., 2015), *P*. *halstedii* (Sharma et al., 2015; Pecrix et al., 2019), *P*. *viticola* (Combier et al., 2019; Dussert et al., 2019), and *B*. *lactucae* (Fletcher et al., 2019). Similar effectors also are present in some *Phytophthora* species (Wood et al., 2019). Intriguingly, recent reports suggest that DM effectors lacking canonical RXLR motif but possessing EER and WY motifs (henceforth referred to as WY-EERs herein) display virulence and avirulence activities during host interaction (Combier et al., 2019; Wood et al., 2019).

It has been suggested that RXLRs from haustoria-forming Peronosporaceae species may be an adaptation to facilitate biotrophy because their expression is induced during pre-infection and biotrophic phases of infection (Whisson et al., 2007; Thines, 2014; Fawke et al., 2015). Therefore, follow up studies that can facilitate the identification of *in planta* expressed effector genes during interaction with the host are essential. To date, there are only two reported studies of gene expression profiling during pathogen-host interactions of haustoria-forming Peronosporaceae species, *Pseudoperonospora cubensis* infecting cucumber (Savory et al., 2012a) and *H. arabidopsidis* infecting *Arabidopsis* (Asai et al., 2014).

*Pseudoperonospora humuli* is an oomycete pathogen that causes DM on hop (*Humulus lupulus*). Symptoms including arrest in shoot development, abortion of developing cones, and defoliation, result in reduced yield, decrease in bittering acids, and even plant death in some cultivars (Ojiambo et al., 2015; Purayannur et al., 2020). DM is perhaps the most important and destructive disease that threatens cone yield and quality in certain production regions (Gent et al., 2009). Management of hop DM is mainly achieved through cultural practices and regular application of fungicides (Gent et al., 2010, 2012). Nonetheless, the pathogen can develop resistance to fungicides and cultivars with resistance to *P. humuli* are rare (Gent et al., 2008, 2020; Gent and Ocamb, 2009). Host resistance is the ideal control method for plant diseases, however, breeding in perennial crops such as hop is a long-term effort (Natsume et al., 2014) and a complicated one because of the narrow genetic base of resistant germplasm. Identification and utilization of effectors to identify germplasm containing *R* genes can accelerate breeding for resistance to DM (Vleeshouwers and Oliver, 2014). Recently, the genome sequence of *P*. *humuli* was published thus expanding the resources in this pathogen (Rahman et al., 2019). In this study we describe the effector repertoire of *P. humuli* with emphasis on the RXLR family. The presence of effector transcripts across different isolates and the expression of core effectors during infection are also presented.

## Materials and Methods

### Pathogen and Plant Material

*Pseudoperonospora humuli* isolate OR502AA, originally isolated from hop in Oregon, was used in this study. For RNA-Seq experiments, the susceptible hop cultivar, Pacific Gem was used. Hop leaves were placed inside square petri dishes (245 mm × 245 mm × 26 mm, Corning, Cat No. 06-443-22) and over wet sterile paper towels. Leaves were drop inoculated using 10 μl of a zoospores solution and incubated at 25°C with a 12 h light/dark cycle in a precision plant growth chamber (Thermo Fisher Scientific, Cat No. PR505755L). Twenty leaf disks were collected with a sterile core borer size 4 (Humboldt Mfg., Co., H9664, Cat No. S50166D) at 2, 3, and 4 days post inoculation (DPI). Leaf samples were ground in liquid nitrogen with a mortar and pestle to a fine powder and stored at −80°C for later RNA extraction.

### RNA Extraction, Library Preparation, and Sequencing of *Pseudoperonospora humuli*

RNA was extracted from a fresh pellet of collected sporangia and from frozen fine leaf powder samples using the Qiagen RNeasy Plant Mini Kit (Qiagen, Cat No. 74904) and submitted to The Genomic Sciences Laboratory at North Carolina State University for library preparation and sequencing in an Illumina NextSeq 500 platform (Illumina, Inc). cDNA libraries of 350 bp insert size were prepared and sequenced as described in Withers et al. (2016). All data generated in this study is available at the Sequence Read Archive (SRA) database under accession PRJNA354153: SRX2363032 (2 DPI), SRX2363027 (3 DPI), SRX2363031 (4 DPI). The predicted coding genes from *P. cubensis* isolate MSU1 (study number SRP011018) reported by Savory et al. (2012b) were used for comparative sequence analysis with *P. humuli* OR502AA. The nuclear genome assembly and annotation of *P. humuli* OR502AA (Rahman et al., 2019) can be found under GenBank accession PRJNA354153, and figshare https://figshare.com/s/5cfeda89bd3d29f3d259 and https://figshare.com/s/35951fc4569554efdc34, respectively.

### Phylogenetic Analysis of Nuclear Genes

A phylogenetic analysis was conducted with a concatenated set of 362 Core Eukaryotic Genes (CEGs) obtained by CEGMA version 2.5 (Parra et al., 2007) following methods by Sharma et al. (2015) with modifications. The 362 single-copy CEG genes from *P. humuli* OR502AA were used to extract homologs shared among 11 oomycete species The oomycete species included: *P. cubensis* MSU1 (Savory et al., 2012b), *P. tabacina* 968-J2 (Derevnina et al., 2015), *P*. *halstedii* BLA4 (Sharma et al., 2015), *H. arabidopsidis* EMOY2 (Baxter et al., 2010), *P. infestans* T30-4 (Haas et al., 2009), *Phytophthora ramorum* PR102a (Tyler et al., 2006), *P*. *sojae* P6497 (Tyler et al., 2006), *Pythium ultimum* BR144 (Levesque et al., 2010), *Albugo laibachii* NC14 (Kemen et al., 2011), and *Saprolegnia parasitica* CBS223.65 (Jiang et al., 2013). All genomes, except for *P. tabacina*, are available at the Fungi and Oomycete Genomics Resources Database^1^ and Ensembl^2^. The *P. tabacina* genome data was obtained by courtesy of R. Michelmore. Multiple sequence alignments were performed using MUSCLE version 3.8.31 (Edgar, 2004) and subsequently concatenated using custom scripts. Maximum likelihood phylogenetic analysis was done with RAxML version 8.2.4 (Stamatakis et al., 2005), with 1000 bootstrap replicates.

### Identification of the Secretome

The 18,656 predicted proteins of *P. humuli* from published genome assembly by Rahman et al., 2019 were evaluated for the presence of signal peptides using SignalP version 2.0 (Nielsen et al., 1997) and for the absence of transmembrane domains with TMHMM version 2.0 (Krogh et al., 2001). Secreted proteins with a SignalP HMM score >0.9, NN cleavage site within 10 and 40 amino acids (aa), and no transmembrane domains found at >40 amino acids away from the starting amino acid methionine, were selected. The gene calls originally inferred from genome assembly for *P. cubensis* MSU1 reported by Savory et al. (2012b) were revised in this study using a BLASTN search against the NCBI nucleotide database with a cutoff sequence coverage of 90% and a percentage of identity of 90% to remove contaminating sequences. The remaining filtered gene calls of *P. cubensis* MSU1 were then analyzed for potential secreted proteins as described above for *P*. *humuli*. Apoplastic effectors were predicted using ApoplastP (Sperschneider et al., 2017). CAZymes were predicted using the dbCAN2 metaserver (Zhang et al., 2018). Effectors were functionally annotated using Blast2GO and BlastP searches against NCBI nr databases using an *e*-value of 1e-0.5.

### RXLR Effector Annotation

The presence of RXLR motifs on secreted proteins was determined with custom scripts as described by Win et al. (2007) with some modifications. RXLR effectors were annotated as such when: the RXLR motif was present within 25 and 110 amino acids, the RXLR position was higher than the NN cleavage site, and the signal peptide length was 10–40 amino acids. We also used the WY domain hidden markov model (HMM) model described by Boutemy et al. (2011) to predict proteins carrying this motif downstream of the RXLR, RXLR-like motif, EER, and EER-like motifs. Proteins were permuted using the script published by Ai et al. (2020) and each script was run separately on the permuted proteins and the total number of motifs predicted was averaged. Proteins were clustered using OrthoFinder version 2.3.3 (Emms and Kelly, 2019). Sub-cellular localization of RXLRs was predicted using DeepLoc version 1.0 (Almagro Armenteros et al., 2017).

### RNA-Seq Analysis

To predict transcript evidence in different isolates of *P*. *humuli*, Illumina RNA-Seq read data obtained from sporangia for OR502AA and 11 other isolates used by Rahman et al. (2019) were aligned to the coding genes of *P. humuli* OR502AA using TopHat2 version 2.0.9 (Trapnell et al., 2009) with 200 bp as the insertion length parameter. Alignments in SAM format were obtained from TopHat2 for gene expression analysis. Absolute read counts were calculated for each gene by using the htseq-count script part of the HTSeq python module (Anders et al., 2014). The reads per kilobase million (RPKM) values were then estimated according to a published method (Mortazavi et al., 2008). To analyze expression data of *P. humuli* isolate OR502AA infecting hop leaf samples at 2, 3, and 4 DPI, SAM alignments from TopHat2 and RPKM values from HTSeq were obtained as described above. To estimate the expression *in planta*, RPKM absolute values were transformed into log2 fold values by dividing the RPKM data from infected leaf tissue by the RPKM values from sporangia of OR502AA (Wagner et al., 2012). The log2 values were used to generate heatmaps using the R package pheatmap. Upset plots were generated using the R package UpsetR (Conway et al., 2017).

### Quantitative RT-PCR

Quantitative RT-PCR was performed using gene-specific oligonucleotides. Tissue was collected and RNA was extracted as described in Sections “Pathogen and Plant Material” and “RNA Extraction, Library Preparation, and Sequencing of *Pseudoperonospora humuli*.” PCR was performed in a CFX96 Touch^TM^ Real-time system (Bio-Rad Laboratories, Inc.) using a PowerUp^TM^ SYBR^TM^ Green Master Mix (Applied Biosystems). Gene-specific primers (Supplementary Table S1) were generated using Primer3 (Untergasser et al., 2012). *P*. *humuli* actin (*Phum_OR502AA_v1_g_08820*), Glycerol-3-phosphate dehydrogenase (*Phum_OR502AA_v1_g_18083*), and ubiquitin-conjugating enzyme E2 (*Phum_OR502AA_v1_g_00443*) were used as reference genes and the values were averaged for calculation. ΔΔCt values were calculated for each time-point using the expression in sporangia as a control by the method published by Livak and Schmittgen (2001) with the slight modification of the initial ΔCt being calculated as ΔCT = CT(reference gene)-CT(target gene) in order to obtain positive values. RT-qPCR was performed three times with independent biological replicates. *P*-values were calculated by a paired *t*-test using the GraphPad Prism 8.00 software for Mac.

## Results

### Phylogenetic Relationship of *Pseudoperonospora humuli* With Other Oomycetes

**FIGURE 1:**
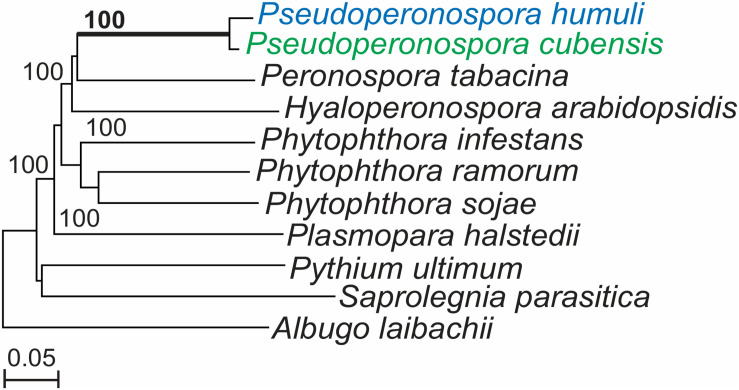
*Pseudoperonospora humuli* and *Pseudoperonospora cubensis* phylogenetic clade generated from conserved nuclear genes. Phylogenetic tree constructed with 362 nuclear core eukaryotic genes (CEGs) sharing homology among 11 oomycetes. CEG genes were aligned with Muscle (Edgar, 2004) and the maximum likelihood tree was generated with RAxML program (Stamatakis et al., 2005), with 1,000 bootstrap replicates.

The genome of *P*. *humuli* showed a 97.8% complete and 98.8% partial completeness based on the coverage of eukaryotic genes (Rahman et al., 2019) described in the CEGMA pipeline (Parra et al., 2007). We generated a nuclear phylogenetic tree of *P*. *humuli* with the 10 other oomycete species listed in Section “Phylogenetic Analysis of Nuclear Genes” using 362 of the core eukaryotic housekeeping genes described by Parra et al. (2007), *P*. *humuli* and its sister species *P*. *cubensis* were clustered in a group with maximum bootstrap support confirming their close relationship (Figure 1). Other DM pathogens such as *P. tabacina*, and the *Arabidopsis* DM *H. arabidopsidis* formed sister groups and separated themselves from the sunflower DM pathogen *P. halstedii* and *Phytophthora* species.

### *Pseudoperonospora humuli* Secretome and Apoplastic Effectors

For *in silico* prediction of the *P*. *humuli* secretome, we used the 18,656 predicted proteins published by Rahman et al. (2019). SignalP v2 was used for the prediction of proteins containing signal peptides since it was noted to be more sensitive for oomycete effectors in comparison to newer versions (Sperschneider et al., 2015b). Using the presence of signal peptide and the absence of a transmembrane domain as criteria, 1,250 secreted proteins were predicted in *P*. *humuli* (Supplementary Table S2). Since apoplastic effectors do not have distinguishing motifs, the machine learning tool ApoplastP (Sperschneider et al., 2017) was used to predict apoplastic effector candidates in the *P*. *humuli* secretome. Among the predicted secreted proteins, 321 proteins were predicted as apoplastic proteins (Supplementary Table S3).

**FIGURE 2:**
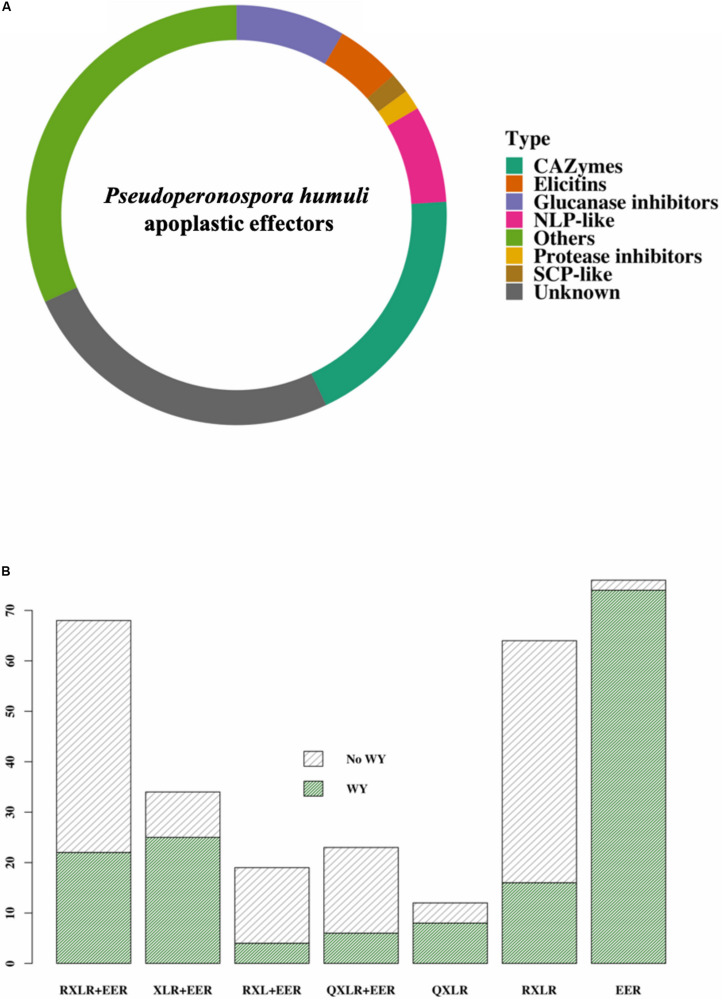
Effectors in *Pseudoperonospora humuli*. **(A)** Classes of apoplastic effectors. Effectors were identified from the secretome by ApoplastP (Sperschneider et al., 2017) and annotated using Blast2Go and BlastP searches. Carbohydrate active enzymes (CAZymes) were identified using the dbCAN2 metaserver (Zhang et al., 2018). [SCP, sperm coat protein; NLPs, necrosis and ethylene-inducing peptide 1 (Nep1)-like proteins]. **(B)** Classes of cytoplasmic effectors. RXLRs were identified using the method described by Win et al. (2007) and WY motifs were predicted by a hidden markov model (HMM) search in HMMER 3.1 (hmmer.org) using a published HMM model (Boutemy et al., 2011).

The predicted apoplastome was classified into known classes of effectors (Figure 2A). CAZymes formed the largest class of apoplastic effectors in *P*. *humuli* (Figure 2A and Supplementary Table S3). Of the five known classes of CAZymes: carbohydrate esterases (CE), glycoside hydrolases (GH), glycosyltransferases (GT), polysaccharide lyases (PL), and auxiliary activity (AA) (Levasseur et al., 2013), *P. humuli* had only CEs, GHs, and AAs (Supplementary Table S3). The *P*. *humuli* apoplastic effector suite also consisted of 32 enzyme inhibitors of which five were protease inhibitors (Figure 2A). All the identified protease inhibitors in *P*. *humuli* belonged to the Kazal-like serine protease inhibitor family. Four of the predicted protease inhibitors in *P*. *humuli* had a single Kazal-like domain (Supplementary Figures S1A,B). The remaining protease inhibitor had five Kazal-like domains (Supplementary Figure S1B). Glucanase inhibitors formed the other abundant class of enzyme inhibitors of which there were 27 proteins in *P*. *humuli*. The *P*. *humuli* glucanase inhibitors had degenerate amino acids at the catalytic triad positions H57, D102, and S195 (Supplementary Table S3).

The *P*. *humuli* apoplastic effector suite had 24 necrosis and ethylene-inducing peptide 1 (Nep1)-like proteins (NLPs). However, the NLPs in *P*. *humuli* had highly degenerate heptapeptide “GHRHDWE” motifs (Supplementary Figure S2). Other predicted *P*. *humuli* apoplastic effector classes were elicitin proteins, sperm coat protein (SCP)-like extracellular proteins, and a myriad of proteins of unknown function (Figure 2A and Supplementary Table S3).

Since *P*. *cubensis* and *P*. *humuli* are sister species, the secretome of *P*. *cubensis* was also analyzed. The published proteome of *P*. *cubensis* (Savory et al., 2012b) was revised using a BLASTN search to remove genes of contaminating microorganisms. Bacterial genes with similarity to common prokaryotes found in the phyllosphere such as Xanthomonadales, Burkholderiales, and Pseudomonadales were found in the *P*. *cubensis* secretome and were removed. Other eukaryotic metazoan contaminants (Laurence et al., 2014) were also removed. Overall, we removed 5,849 gene contaminants out of 23,522 (Supplementary Table S4) coding genes predicted for *P. cubensis*, leaving the final *P*. *cubensis* protein count as 17,673. Of these, 941 genes were predicted to be secreted proteins using the same pipeline that was employed for *P*. *humuli* secretome prediction (Supplementary Table S5). ApoplastP predicted 216 proteins as putative apoplastic effectors (Supplementary Table S6). The numbers of the apoplastic effector classes were reduced in *P*. *cubensis* as compared to *P*. *humuli* (Table 1). Cluster analysis of apoplastic effectors of the two species showed that there were 122 clusters common to *P*. *humuli* and *P*. *cubensis*. *P*. *humuli* had more singletons than *P*. *cubensis* (Supplementary Figure S3A).

**TABLE 1:** Comparison of secretome and effector families in *Pseudoperonospora humuli* and *Pseudoperonospora cubensis.*

**Description**	***Pseudoperonospora humuli* (OR502AA^a^)**	***Pseudoperonospora cubensis* (MSU1^b^)**
Predicted proteins	18,656	17,673
Secreted	1,250	941
CAZymes	61	39
Glucanase inhibitors	27	5
Protease inhibitors	5	3
NLPs	24	14
Total RXLR and RXLR-like	296	72
EER/EER (like)+WY	74	12
CRN total (secreted)	53 (1)	15 (0)

*^*a*^Published annotation of *P*. *humuli* (Rahman et al., 2019) was used to predict the secretome and effectors. ^*b*^Published annotation of *P*. *cubensis* (Savory et al., 2012b) was filtered to remove contaminants and was used to predict the secretome and effectors (this study). NLPs, necrosis and ethylene-inducing peptide 1 (Nep1)-like proteins.*

### Cytoplasmic Effectors in *Pseudoperonospora humuli*

Rahman et al. (2019) predicted a total of 189 putative RXLR-like effector candidates and 49 CRinkling and Necrosis (CRN)-like candidates (with or without signal peptides) from *P*. *humuli*. We re-examined the genome of *P*. *humuli* using a comprehensive *in silico* string search and similarity analysis to expand the *P*. *humuli* cytoplasmic effector repertoire. Our analysis revealed a total of 296 RXLR-EER and RXLR-EER-like cytoplasmic effector candidates in the *P*. *humuli* secretome, including those proteins that contained non-canonical RXLR and/or EER motifs (Figure 2B and Supplementary Table S7). Among the predicted RXLR-like effector candidates, a large number of these effectors contained RXLR and EER motifs (Figure 2B). Several of these effectors contained non-canonical EER motifs (Supplementary Table S7). There were 35 QXLR motif-containing effectors in *P*. *humuli* including some that contained only a QXLR motif and lacked an EER (Figure 2B). Degeneracy in the RXLR motif was observed in some effectors, with changes in the first and the second arginine residues. Proline (P) and lysine (K) were common substitutes for R in the first position while glutamine (G) and K were common in the second position (Supplementary Table S7).

We used the previously published HMM model (Boutemy et al., 2011) to search for WY domains in the identified RXLRs in *P*. *humuli*. 154 out of the identified 296 RXLRs had one or more WY domains. An interesting observation was the high number of WY-EERs in *P*. *humuli* (Figure 2B). Of the 154 WY domain-containing effectors, 74 were WY-EERs (Figure 2B and Supplementary Table S7). It has been proposed previously (Wood et al., 2019) that an HMM search for WY domains in the whole secretome is an important step in oomycete effector prediction to identify additional effectors that are not revealed in RXLR-EER string searches. However, an HMM search of the *P*. *humuli* secretome failed to reveal additional WY effector candidates.

The sub-cellular localization of the 296 RXLR candidates was predicted using Deeploc v1 (Almagro Armenteros et al., 2017). There were 140 proteins that were predicted to be localized to the cytoplasm and 98 that were predicted to localize to the nucleus. There were a few proteins that had predicted localization to mitochondria, plastid, and peroxisome (Supplementary Table S8). In contrast to *P*. *humuli*, the secretome of the sister species *P*. *cubensis* had only 72 RXLR and RXLR-like proteins (Supplementary Table S9). There was a considerable reduction in number of RXLR effectors in *P*. *cubensis* as compared to *P*. *humuli* (Table 1). Gene orthology analysis of identified effector candidates in *P*. *humuli* and *P*. *cubensis* revealed 20 clusters that were common to both species (Supplementary Figure S3B).

A permutation test was performed to estimate the false discovery rate (FDR) of scripts used for analysis. The first 150 residues of the *P*. *humuli* and *P*. *cubensis* secretomes were permuted 150 times and the average number of motifs predicted was calculated. The FDRs for N-terminal single-motif scripts were higher than those for double-motif scripts as expected. FDRs were higher in *P*. *cubensis* than *P*. *humuli* with the scripts for QXLR motifs showing a higher FDR (Supplementary Table S10).

To identify members of the CRN (CRinkling and Necrosis) family of effectors in *P*. *humuli*, an HMM search was performed using a previously published HMM model (Haas et al., 2009). The *P*. *humuli* secretome had a single candidate that possessed an LFLAK-like (LYLARK) and an HVLVXXP-like motif. On the other hand, an HMM search on the 941 *P*. *cubensis* secreted proteins produced no hits. Since the low number of CRN effectors was surprising, an HMM search was performed on all 18,656 predicted *P*. *humuli* proteins, which resulted in 62 hits of which nine had no LFLAK or HVLVXXP motifs. Among the remaining 53 CRNs, 20 had both LFLAK-like and HVLVXXP-like motifs (Table 1 and Supplementary Table S11). Apart from these, there were 24 proteins with only an LFLAK-like domain and nine with only an HVLVXXP-like motif. However, only one of them had a SignalP HMM probability >0.9. *P*. *cubensis*, on the other hand had only 15 CRNs with an LFLAK-like and/or or an HVLVXXP-like motif but none had a predicted signal peptide (Table 1 and Supplementary Table S12).

### Sporangial Expression of Effectors in Different *Pseudoperonospora humuli* Isolates

**FIGURE 3:**
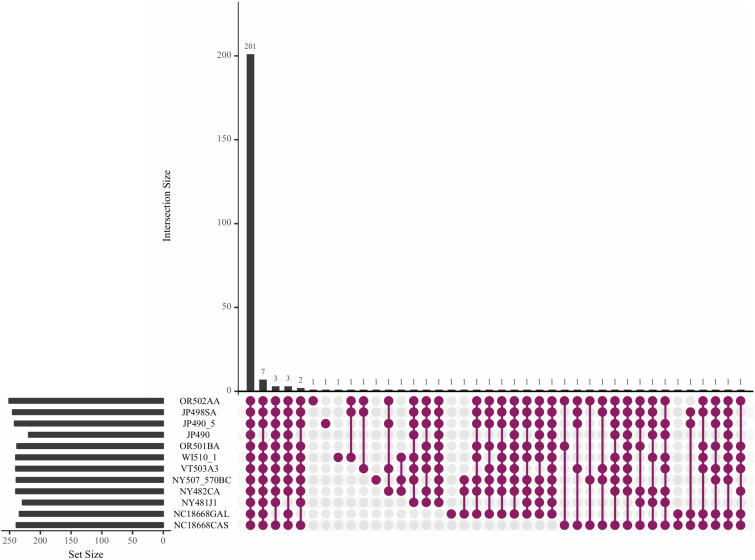
Upset plot showing the trend of effector transcripts in the sporangia of *Pseudoperonospora humuli* isolates. RNAseq reads from sequenced sporangia of different isolates were mapped to *P*. *humuli* gene calls (Rahman et al., 2019) using TopHat version 2.0.6 (Trapnell et al., 2009). Reads were counted using the HTSeq python module (Anders et al., 2014) and reads per kilobase million (RPKM) values were calculated. An RPKM > 0 was used to indicate the presence of a transcript. The plot was generated using the R package UpsetR (Conway et al., 2017).

Core effectors that are expressed across different isolates may be good candidates for effector-assisted breeding. For this reason, we looked for RXLRs that have conserved transcript evidence in different *P*. *humuli* isolates. RNA-Seq performed on sporangia of the *P*. *humuli* isolate OR502AA and eight others used by Withers et al. (2016) were used to study the presence of transcripts of the predicted effector-encoding genes. In addition, we also used the three isolates NY507570BC, NC18668CAS, and NC18668GAL used by Rahman et al. (2019). Of the total 18,656 predicted genes in *P*. *humuli*, we found transcript evidence with an RPKM value > 0 for 10,536 genes in all the sequenced isolates (Supplementary Figure S4A and Supplementary Table S13). Of the 1,250 predicted secreted proteins, 754 showed transcript evidence (Supplementary Figure S4B), out of which, 171 were apoplastic effectors (Supplementary Figure S5). Two hundred one out of the predicted 296 RXLR genes exhibited transcript evidence in all the tested isolates (Figure 3). The expression level of the 201 common effectors was analyzed across the isolates (Supplementary Figure S6). Drastic differences in sporangial expression were not observed across the isolates tested. We investigated differences in transcript presence/absence based on the geographical location from which sporangia were collected. There were 85 genes that did not show transcript evidence in the two isolates collected from North Carolina (Supplementary Figure S4A). Out of these, two were genes coding for secreted apoplastic effectors (Supplementary Figures S4A, S5). Apart from this, we did not observe any notable similarities or differences in isolates collected from different cultivars or geographical locations.

### *Pseudoperonospora humuli* Effectors That Are Expressed During Infection

The susceptible hop variety Pacific Gem was infected with the *P*. *humuli* isolate OR502AA and tissue was harvested at 2, 3, and 4 DPI and sequenced for expression profiling. Out of the 18,656 predicted genes, 1,381 were upregulated (log2 fold value ≥ 2) at all time-points post inoculation (Supplementary Table S14). There were 197 genes that were upregulated only at 2 DPI out of which 23 were uniquely expressed (log2 value 0 at 3 DPI and 4 DPI). Likewise, out of the 168 genes upregulated at 3 DPI, 57 were uniquely expressed and of the 190 genes upregulated at 4 DPI, 82 were uniquely expressed. Among the 321 predicted apoplastic effectors, 64 showed upregulation at all time-points (Supplementary Figure S7 and Supplementary Table S14). Glucanase inhibitors as a class were notable among the upregulated apoplastic effectors. It was also interesting to note that seven among the 11 upregulated glucanase inhibitors showed transcript presence in all the *P*. *humuli* isolates tested, suggesting the importance of this class of apoplastic effectors in infection (Supplementary Figure S7 and Supplementary Table S14).

The expression pattern of cytoplasmic effectors at different time-points was also analyzed. The single CRN gene with a predicted signal peptide (*Phum_OR502AA_v1_g_03069*) did not show upregulation at any time-point. Out of the 296 predicted RXLR effectors, 62 were upregulated (log2 values ≥ 2) at all time-points. It was interesting to observe that most of the expressed effectors showed consistent levels of expression across the time-points tested (Supplementary Figure S8). There were 6, 4 and 6 upregulated effectors each at 2 DPI, 3 DPI, and 4 DPI, respectively (Supplementary Figure S8 and Supplementary Table S14). The gene *Phum_OR502AA_v1_g_16070* was uniquely expressed at 4 DPI (Supplementary Figure S8 and Supplementary Table S14). Since *P*. *humuli* showed an expansion of WY-motif containing effectors with EER or EER-like domains, we were specifically interested in the expression profile of that class. Of the 74 EER-like effectors with one or more WY motifs, 10 were upregulated at all time-points (Supplementary Table S14).

There were 75 core effectors in *P*. *humuli* that were upregulated during infection and were present in all the isolates tested (Supplementary Table S15). This list included both apoplastic and cytoplasmic effectors. RXLR-EERs with no WY domains and glucanase inhibitors were the largest class in this list.

**FIGURE 4:**
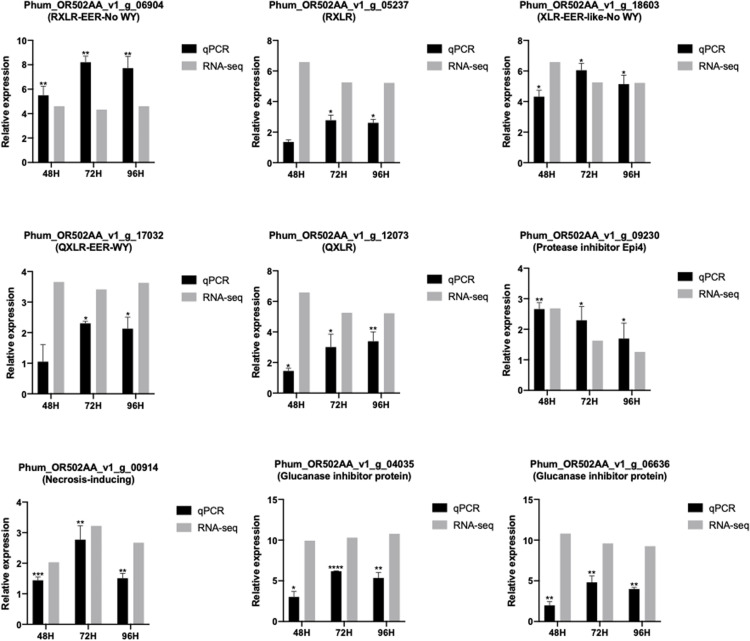
Comparison of RNAseq and RT-qPCR expression values of selected effector genes in different time-points of *Pseudoperonospora humuli* infection of hop. RNA-seq expression levels are depicted in log2 values of reads per kilobase million (RPKM) values. RT-qPCR expression levels are represented as mean ΔΔCT values from three independent biological replicates. *P-value < 0.05, **P-value < 0.01, ***P-value < 0.001, ****P-value < 0.0001. *P*-values were calculated using a paired *t*-test in the GraphPad Prism 8.00.

The expression patterns of selected genes were validated by quantitative RT-PCR. Randomly selected RXLR genes and apoplastic effector-encoding genes were analyzed for their expression patterns. CRN genes were not selected for qPCR since the only CRN with a predicted signal peptide did not show elevated expression in RNA-seq data. All genes showed significant upregulation at all time-points with patterns comparable to those seen in the RNA-seq data (Figure 4).

## Discussion

### Effector Content in *Pseudoperonospora humuli* and *Pseudoperonospora cubensis*

Our analysis predicted that 1,250 out of 18,656 (6.69%) proteins in *P*. *humuli* were secreted as compared to 941 out of 17,673 (5.32%) in *P. cubensis*. Despite similar secretome sizes between *P*. *humuli* and *P*. *cubensis*, there was an increased number of predicted effector proteins in *P*. *humuli*. The apoplastic effector content in *P*. *humuli* was much higher than in *P*. *cubensis* with a notable increase in glucanase inhibitors and CAZymes. The most astonishing increase in the number of effectors between *P. humuli* and *P. cubensis*, however, was observed in the RXLR class of effectors, especially in the number of WY-EERS. Also, despite the close phylogenetic relationship between *P*. *humuli* and *P*. *cubensis*, the number of orthologous RXLRs were few. This lack of orthology between species of the same genus is not surprising considering the high divergence in the RXLR family that has previously been reported in *Phytophthora* spp., with *P*. *sojae* and *P*. *ramorum* having a low number of shared orthologs (Jiang et al., 2008). Moreover, *P*. *cubensis* has most likely arisen due to a host jump from *P*. *humuli* and it has been suggested that loss of effectors required for hop colonization has possibly occurred in *P*. *cubensis* following the pathogen’s adaptation to a new host (Runge and Thines, 2012). It is also interesting to note that gene loss associated with host jumps may be due to the loss of genes that do not have targets in the new host (Sharma et al., 2014; Thines, 2019). There was a large reduction in the number of RXLRs in *P*. *cubensis* in our analysis from the earlier predicted 271 RXLRs (Savory et al., 2012a). However, differences in the pipelines used for genome annotation and effector prediction in the two taxa cannot be ruled out. Moreover, oomycete effectors are known to reside in repeat-rich regions (Raffaele and Kamoun, 2012). Due to the inherent difficulties associated with assembling repeat-rich regions with short-read sequencing technologies like Illumina, some effector genes may be missing from the prediction performed in this analysis. Nevertheless, the number of effectors predicted in *P*. *humuli* is comparable to the numbers found in other sequenced DM pathogens (Derevnina et al., 2015; Sharma et al., 2015; Dussert et al., 2019).

The variation in numbers between *P*. *humuli* and *P*. *cubensis* could also be attributed to our initial filtering for contaminants in the *P*. *cubensis* proteome. The presence of contaminants is not surprising in next generation sequencing data from biotrophic pathogens like *P*. *cubensis* and *P*. *humuli* (Laurence et al., 2014; Rahman et al., 2019). The finding of bacterial sequences in the *P*. *cubensis* proteome affirms the need for specialized laboratory protocols that account for the phyllosphere microbiome and stringent bioinformatics filtering in initial steps when dealing with the genomic data of biotrophic pathogens (Klein et al., 2019). With the range of sequencing technologies now available, resequencing the *P*. *cubensis* genome with clean material and the use of *in silico* methods to remove contaminants might result in more accurate RXLR predictions.

The permutation test revealed a higher FDR for proteins with positional constrained N-terminal single-motif scripts, especially for proteins with the prediction of QXLRs motifs. It is therefore important to validate the veracity of effector proteins containing degenerate RXLR motifs such as QXLR that lack a downstream EER motif with experiments such as quantitative RT-PCR before they are used for further functional studies.

### Apoplastic Effectors of *Pseudoperonospora humuli*

In this study, we predicted the putative apoplastic effectors in the *P. humuli* secretome using the tool ApoplastP (Sperschneider et al., 2017). Unlike oomycete cytoplasmic effectors such as RXLRs and CRNs, apoplastic effectors do not have conserved motifs that can be used for *in silico* prediction. Hence, apoplastic effectors have been traditionally discovered through experimental methods like proteomics (Delaunois et al., 2014) and microscopic analysis (Doehlemann and Hemetsberger, 2013), which are time consuming. ApoplastP has limitations as an *in silico* prediction tool, hence, predicted apoplastic effectors need to be experimentally verified before downstream applications.

*Pseudoperonospora humuli* proteins predicted to be secreted to the host apoplast included known enhancers of necrotrophy and elicitors of microbe associated molecular pattern (MAMP) responses. For example, NLPs belong to a gene family that induces cell death and their role has been indicated in the switch from the biotrophic to necrotrophic phase in *P*. *infestans* (Qutob et al., 2002). The presence of NLPs in a biotrophic pathogen was surprising, however, they have been previously predicted in other DM pathogens like *H*. *arabidopsidis* (Baxter et al., 2010; Cabral et al., 2012), *P*. *tabacina* (Derevnina et al., 2015), and *P*. *halstedii* (Sharma et al., 2015). Similar to NLPs in *H*. *arabidopsidis*, the *P*. *humuli* NLPs also had highly degenerate catalytic domains suggesting that they are non-cytotoxic proteins (Cabral et al., 2012). Unlike NLPs in *H*. *arabidopsidis*, which are expressed during infection (Cabral et al., 2012), only three of the 24 *P*. *humuli* genes (*Phum_OR502AA_v1_g_00914*, *Phum_OR502AA_v1_g_18234*, and *Phum_OR502AA_v1_g_19197*) showed elevated expression *in planta*. However, variation in the time-points tested could have contributed to this discrepancy since the highest expression in *H*. *arabidopsidis* was observed during the early stages of infection (Cabral et al., 2012). Even though the importance of NLPs in DM pathogens is yet to be defined, their conservation among the different species suggests a possible role in pathogenicity. In *H*. *arabidopsidis*, NLPs also act as MAMPs and trigger immune response (Oome et al., 2014), which could be a possible reason for their predicted secretion and localization to the apoplast. Elicitins are another class of pathogen-secreted proteins that act as MAMPs (Derevnina et al., 2015; Raaymakers and den Ackerveken, 2016). Despite their negative role in pathogenicity, MAMPs like elicitins are important in disease resistance as evidenced by the successful transfer of elicitin-induced resistance against late blight to cultivated potato (Du et al., 2015). For this reason, we deemed it necessary to include proteins like NLPs and elicitins in the *P*. *humuli* effectorome, keeping with the definition of effectors as any pathogen-secreted molecules that induce or suppress plant responses (Vleeshouwers and Oliver, 2014).

CAZymes have been observed in several species of *Phytophthora* (Ospina-Giraldo et al., 2010; Brouwer et al., 2014) and *Pythium* (Zerillo et al., 2013) and their role in pathogenicity by the degradation of the cell-wall has been discussed in oomycete (Ospina-Giraldo et al., 2010; Zerillo et al., 2013) and fungal (Lyu et al., 2015) pathogens. The presence of CAZymes in the genome of pathogens alone does not implicate their role in pathogenesis since they could also be involved in the degradation and/or modification of the pathogen cell wall. However, the inclusion of CAZymes in the predicted apoplastic effector repertoire of *P*. *humuli* and the elevated expression of some members of the class indicates their possible role in pathogenesis. It is interesting to note that in previous studies CAZymes had shown elevated expression in *P*. *cubensis* during different stages of infection (Savory et al., 2012a).

The plant apoplast is a barrier that the pathogen has to overcome in order to establish infection. Host-secreted endo-β-1,4-glucanases induce the release of glucan elicitors that are recognized by host cell-surface receptors to activate immunity (Rose et al., 2002). Glucanase inhibitors, which are serine protease homologs that inhibit secretion of the plant endo-β-1,4-glucanases, are abundant in *P*. *infestans* (Damasceno et al., 2008) but have not been well-described in other sequenced DMs. However, the high number of glucanase inhibitors in *P*. *humuli* and their elevated expression during infection implicates their role in pathogenesis. Protease inhibitors were also identified in *P. humuli*. Three of the predicted protease inhibitors in *P*. *humuli* had a single Kazal-like domain that shared similarity to the EPI1b domain of the *P*. *infestans* EPI1 protein (Tian et al., 2004). However, none of these three proteins showed elevated expression during infection. The protease inhibitor with five Kazal-like domains, on the other hand, showed transcript evidence in all the isolates and elevated expressions during infection. The other two protease inhibitors showed elevated expression during infection and the expression pattern of *Phum_OR502AA_v1_g_09230* was confirmed using quantitative RT-PCR as well.

### Cytoplasmic Effectors in *Pseudoperonospora humuli*

The oomycete cytoplasmic RXLR and CRN effector classes are well-documented mainly due to their modular nature. CRNs are an ancient class of effectors that have been identified across phylogenetically diverse oomycete species (Schornack et al., 2010). The CRNs are a large family of effectors in *P*. *infestans* (Haas et al., 2009). In *P*. *humuli*, however, only a single CRN was identified in the secretome. A search for CRNs in the total proteome yielded more CRN candidates that contained LFLAK-like and/or HVLVXXP motifs, however these did not contain signal peptides. This trend has been observed in other DM CRNs (Derevnina et al., 2015; Sharma et al., 2015; Fletcher et al., 2018). The fewer CRN content in DM pathogens could indicate an adaptation to biotrophy since CRNs are known to induce necrosis, a trait that is not conducive for biotrophy. Intriguingly, oomycetes are known to use unconventional secretion pathways to secrete CRNs lacking a classical signal peptide (Meijer et al., 2014), suggesting that the identified CRNs in *P*. *humuli* might be secreted even though they lack a classical secretion signal. Unfortunately, there are no existing reliable algorithms for the prediction of unconventionally secreted proteins in fungi and oomycetes (Sperschneider et al., 2015a).

Effectors containing the N-terminal RXLR-EER motif are the best-understood pathogenicity factors of *Phytophthora* spp. and DM pathogens. *P*. *humuli* had a total of 296 RXLR and/or EER-domain containing proteins. This number includes proteins containing degenerate RXLR or EER domains. A non-canonical QXLR motif has been observed among cytoplasmic effectors in *P*. *cubensis* (Tian et al., 2011; Savory et al., 2012a) and *B*. *lactucae* (Wood et al., 2019). The high number of QXLRs in *P*. *humuli* reiterates their significance in DM pathogens. Some RXLRs are distinguished by the presence of a C-terminal α-helical fold known as the WY domain (Jiang et al., 2008; Boutemy et al., 2011). Traditionally, prediction of RXLR effectors had been based on the presence of the RXLR and the EER motif. However, recently, the presence of WY domain containing EER effectors have been identified in DM pathogens (Derevnina et al., 2015; Combier et al., 2019; Wood et al., 2019). The expansion of WY-EER effectors in *P*. *humuli* further affirms the importance of this class in DM pathogens. Intriguingly, WY-EERs form the largest class of effectors in *P*. *humuli* outnumbering even the classical RXLR-EERs. In the light of the overrepresentation of WY-EERs in *P*. *humuli*, we reiterate previous recommendations to include HMM searches for WY domains as a key criterion to identify effectors in oomycetes and especially DM pathogens (Derevnina et al., 2015; Wood et al., 2019).

The *P*. *humuli* RXLR-EER gene *Phum_OR502AA_v1_g_06904* showed transcript evidence in all the 12 isolates and elevated expression in RNA-seq and RT-qPCR. A BLASTp search did not show obvious homology to any known protein except for a weak similarity (25.8 percentage identity) to a bacterial shikimate kinase. The lack of homology to known RXLRs is not surprising since RXLRs are a class of diverse proteins that show a high level of sequence divergence outside of the conserved N-terminal RXLR and EER motifs (Win et al., 2012). Despite the lack of an apparent function, the core nature of *Phum_OR502AA_v1_g_06904* across the *P*. *humuli* isolates and its high expression during infection may indicate a role during the host-pathogen interaction making it a good candidate for downstream functional analysis.

### Core Effectors of *Pseudoperonospora humuli*

Natural sources of DM resistance in hop are rare and for this reason, identifying new sources of resistance is a priority (Woods and Gent, 2016). However, breeding for resistance to DM is a laborious and time-consuming process, especially in a perennial crop sensitive to inbreeding depression (Henning et al., 2004). Pathogen effectors can be used to accelerate disease resistance through effector-assisted breeding (Vleeshouwers and Oliver, 2014) and loss of susceptibility breeding (Pavan et al., 2010). However, core effectors that are conserved across different isolates need to be validated in order to make an informed choice on the candidate effector that is selected. Effectors that are present in various pathogen isolates and also expressed during infection are considered core effectors, which are key to identifying broad spectrum and durable plant resistance genes (Jones et al., 2014; Vleeshouwers and Oliver, 2014). The *P*. *humuli* effectorome contained effectors that exhibited transcript evidence in all *P*. *humuli* isolates and showed enhanced expression during infection. We propose that these core effectors are ideal candidates for downstream functional analysis aiming toward the identification of robust sources of resistance to DM in hop.

## Data Availability Statement

The datasets generated for this study can be found in the National Center for Biotechnology Information database under the Bioproject number PRJNA354153.

## Author Contributions

LQ-O, SP, and LC conceived and designed the experiments and wrote the manuscript. SP, LC, MB, KC, and DG performed the experiments. SP, LC, MB, and KC analyzed the data. LQ-O, DG, and KC contributed reagents, materials, and analysis tools. All authors contributed to the article and approved the submitted version.

## Conflict of Interest

MB is employed by the company Ball Horticultural Company, West Chicago, IL, United States.

The remaining authors declare that the research was conducted in the absence of any commercial or financial relationships that could be construed as a potential conflict of interest.

## Footnotes

1 https://fungidb.org/fungidb/

2 http://protists.ensembl.org/index.html

## Supplementary Material

TABLE S1 List of genes used for RT-qPCR and their primer sequences.

TABLE S2 Secretome annotations and amino acid sequences *Pseudoperonospora humuli*.

TABLE S3 Predicted apoplastic effectors in *Pseudoperonospora humuli*, their annotation, SignalP HMM score, signal peptide position, and amino acid sequences. The different classes are separated in different sheets. The degenerate amino acids in the catalytic triad of glucanase inhibitors are also presented.

TABLE S4 List of *Pseudoperonospora cubensis* predicted contaminant coding genes (CDSs) identified using BLASTN search against NCBI nr database.

TABLE S5 Secretome annotations and amino acid sequences *Pseudoperonospora cubensis.*

TABLE S6 Predicted apoplastic effectors in *Pseudoperonospora cubensis*, their annotation, SignalP HMM score, signal peptide position, and amino acid sequences. The different classes are separated in different sheets.

TABLE S7 RXLR effector annotations and amino acid sequences for *Pseudoperonospora humuli*.

TABLE S8 Predicted sub-cellular localizations of RXLR effectors in *Pseudoperonospora humuli*. Predictions were made using DeepLoc-1.0 (Almagro Armenteros et al., 2017).

TABLE S9 RXLR effector annotations and amino acid sequences for *Pseudoperonospora cubensis*.

TABLE S10 Average number of motifs in permuted sequences and secretome in *Pseudoperonospora humuli* and *Pseudoperonospora cubensis*.

TABLE S11 CRN effectors of *Pseudoperonospora humuli.* Motif sequences and positions of LFLAK and HVLVXXP motifs are presented. Single protein with predicted signal peptide are highlighted.

TABLE S12 CRN effectors of *Pseudoperonospora cubensis.* Motif sequences and positions of LFLAK and HVLVXXP motifs are presented.

TABLE S13 Reads per kilobase million (RPKM) values of RXLR effectors in the sporangia of different *Pseudoperonospora humuli* isolates.

TABLE S14 Log2 transformed reads per kilobase million (RPKM) values of *Pseudoperonospora humuli* genes during infection on hop.

TABLE S15 List of core effectors in *Pseudoperonospora humuli.*
