# Supplementary material for: The Effector Repertoire of the Hop Downy Mildew Pathogen Pseudoperonospora humuli
Source: Front Genet. 2020 Aug 11;11:910. doi: 10.3389/fgene.2020.00910 (PMC7432248; doi:10.3389/fgene.2020.00910)
Supplement: Supplementary file 16 [file Data_Sheet_1.PDF]

(A)

```
P_infestans                 CSTVCPDVELPVCGSNRVRYGNPCELRIAACEHPELNIVEDSGKAC 46
Phum_OR502AA_v1_g_12829 CDQVCDTLYDPVCGTDGVTYANDCAFSVAQCKKSDLRVLAEGEC-- 44
Phum_OR502AA_v1_g_07312 CSNFCNRDYVPVCGSDGVTYGNKCVFEYASCMNSTISKASDGEC-- 44
Phum_OR502AA_v1_g_10993 CDDNCERDYMPVCDSNGDKYDNMCLFEFAQCKNSTISAAPC----- 41
```

(B)

```
>Phum_OR502AA_v1_g_19576
MKFALGLLLAAVAVTDTFGESLTPNGPPITGLGPTDTSSQNLTVQQAIQDLADQSSGNGN
ENINVINPQTLPVQNPADIAGLGPTTPLQQNQQNQQNQQQPQGTPTTNTLVINPNQKPFT
PSTLEGLGPQTPAGSNQNIQNGIKQLAGPPASTPTNNDNFHNIDPRTLPVVDPKCQNRQG
PVTQSGQFTQEEMEKLLRESADGCDGQPRNCDRMCPEVYEPVCGSDYKVYSNSCFLSLAA
CQDPGITQVEDDACSDQPRNCDRMCPDVYEPVCGSDYKVYSNSCFLDLAACQDPGITQVE
DDACSDQPRNCDRMCPDVYEPVCGSDYKVYSNSCFLSLAAXXXXXXXXXXXXXXXXXXXX
XXXXXXXXXXXXXXXXXXXXXXXXXXXLAACQDPGITQVEDDACSDQPRNCDRMCPDVYE
PVCGSDYKVYSNSCFLDLAACQDPGITQVEDDACSDQPRNCDRMCPDVYEPVCGSDHIRY
SNSCFLNLAACQDPGITQVEDDACSDPATGIMQN

>Phum_OR502AA_v1_g_09230
MKFALGLLLAAAAIAGTFGESTTMIPPPSSPPLFTGLGPQTPAGDNPQIRQGIEQLAGPP
APTPTNNDNFQNVDPRTLVDPKCQNGTVTVPQSGQFSLYEFNKILEEYVEGCNGQTPFCD
RMCPDIYQPVCGNDNKIYTSSCNLILAACHDPIRRIRLTDTC
```

Supplementary Figure S1. Protease inhibitors in *Pseudoperonospora humuli***.** (A) Alignment of the three Kazal-like protease inhibitors that show similarity to the Epi1b domain of the *Phytophthora infestans* Epi1 (blue); (B) The remaining two Kazal-like protease inhibitors with 5 and 1 Kazal-like domains (highlighted), respectively.

```
P.infestans_NPP1          QKAGIKFKPQIHISN-GCHPYPAVDADGNTSGGLNPT--GSSSAGCKGSG----YGSQIY    93
Phum_OR502AA_v1_g_06621   EKCAVAFKPALTVVS-GCYPYPAVNAEGQTNLGDRMFK---NNTNCGKPA----NGSQIY    90
Phum_OR502AA_v1_g_19197   EKCAVAFKPALAVFS-GCYPYPAVNAAGQTSLGNRLFK---EDALCGMPA----NGSQVY    90
Phum_OR502AA_v1_g_18324   DQSGVMFKPTLLSTT-GCQSYAAIDAEGRTNEGAEPV--GMLYEECEGSD----HGSQVY    92
Phum_OR502AA_v1_g_01714   DRSGVWFKPSLLDVS-GCRPYPVVDASGRTGAGNEEG--IPRLERCQGRRAEKSPPPQVY    97
Phum_OR502AA_v1_g_10030   EKSGVRFKPSLLVTA-GCQPYPAVDASGQTSAGNEKQ--DPSYQGCSSST----HRSQVY    93
Phum_OR502AA_v1_g_14034   EKSGVRFKPSLLVSS-GCEPYPAVDASGRTSTGNEAG--DVSEKGCYGLT----HGFQVY    93
Phum_OR502AA_v1_g_01522   DKSGVRYKPSLIVTK-GCHSYPAVDASGRTSAGDEKA--EPFNNGCLDPT----QGDQVY    93
Phum_OR502AA_v1_g_05939   QKSGVRFKPSLIVTS-GCHPYAAVDASGRTSAGNEAG--DSMNQLCLYPT----LGAQVY    93
Phum_OR502AA_v1_g_18670   DKSGVKFKPSLIVTS-GCHPYPAVDASGRTSAGNEGG--ASYDYGCKGPQ----RDSQVY    93
Phum_OR502AA_v1_g_19463   DISGLAYQPTLLVSG-GCVPYPAVDAKGDTSGGLVGNDEAFPYTSCSGSR----HGSQVY    95
Phum_OR502AA_v1_g_08265   DRSGVRYQPSLTVTG-GCLPYPAVNARGETSGGFIGDDESFITLGCEGSE----HGSQVY    92
Phum_OR502AA_v1_g_05046   DKSGVRYQPSLTVNG-GCLPYPAVDASGATSNGLPIAYGDLPNSGCGIQE----RGAQVY    94
Phum_OR502AA_v1_g_00914   DKSGVWYQPSLTVNL-GCLPYPAVDASGDTSGGLEPSSESVSSDGCGGEG----SESQVY    94
Phum_OR502AA_v1_g_08901   DKSGVWYKPRLTVDL-GCLPYPAVDASGDTSGGIKTSPGSLSFIGCGGSK----HGSQVY    94
Phum_OR502AA_v1_g_14668   DKSGVWYKPRLTVNL-GCLPYPAVDASGDTSGGLAIGPGGLPSTDCGGTR----HGSQVY    94
Phum_OR502AA_v1_g_02635   EKSAVKYKPQLHISS-GCYPYPAVQADGAVSGGLGLP--IFPDGKCKGSE----LGSQIY    92
Phum_OR502AA_v1_g_07619   EKSAVKYKPQLHISS-GCYPYPAVQADGAVSGGLGLP--IFPDGKCKGSE----LGSQIY    92
Phum_OR502AA_v1_g_07620   EKSAVKYKPQLHISS-GCYPYPAVQADGAVSGGLGLP--IFPDGKCKGSE----LGSQIY    92
Phum_OR502AA_v1_g_19643   EKSAVKYKPQLHISS-GCYPYPAVQADGAVSGGLGLP--IFPDGKCKGSE----LGSQIY    92
Phum_OR502AA_v1_g_11522   EKLAIKHKPQIVIAY-GCDPYPAVAANGDYSGGLKFS--GDHDGECKGSG----LGSQIY    93
Phum_OR502AA_v1_g_18234   QKSAVKFKPLLAIGPDECWPHTAVTVDGKVSSGLPTA-----ESNCLT------FQGQVY    107
Phum_OR502AA_v1_g_08027   EKSAIKFKPRLTIRPGRCYPHAAVTVDGKISSGLSQE-----NLECGT------VQGQVY    106
Phum_OR502AA_v1_g_13035   EKSAIKFKPRLTIRPGRCYPHAAVTVDGKISSGLSQE-----NRECVT------VQGQVY    106
Phum_OR502AA_v1_g_02174   AHSGVRFKPSLLVTA-GCEPYPAVDAKGQTGAGNEDG--ALYRQKCAGIG----HASQVY    93
```

```
P.infestans_NPP1          GRVATYNGVFAIMYSWYFPKDSPL------TGLGHRHDWEHVVVWVDDIKLN-SPSVIAV    146
Phum_OR502AA_v1_g_06621   GRSEWHKELWANMYSWYHPKDLTVENEQPGYENSHVHEWTSIVVWVTNPLP--PNKISAV    148
Phum_OR502AA_v1_g_19197   GRSEWHKELWANMYSWYHPKDQTADGNEIKYVGSHVHEWTSIVVWVTNPLP--PNKISAV    148
Phum_OR502AA_v1_g_18324   GRAMWHKTLWAIMYAWYFPKNQPFA----AGDTGHRHDWSSIVIWIDNPKVL-KPRIIGV    147
Phum_OR502AA_v1_g_01714   GRAIWHRDQWAIMYAWYYPLNLYN--L--FYKPHLPHYWVSVVVWITNPEV--EPKIIGV    151
Phum_OR502AA_v1_g_10030   GRVMWHRDQWAFMYAWYFPMDKPDSGS--EETPFFSHDWTSVVVWITNPEV--EPKIIGV    149
Phum_OR502AA_v1_g_14034   GRAMWHRNRWAIMYAWYLPMDMPG-----EVRPIFSHDWPSVVVWIDNPAVP-EPKIIGV    147
Phum_OR502AA_v1_g_01522   GRAIWHKGRWAIMYAWYFPMDNAG-----WYKPFFSHDWASIVVWIDNPAVP-EPTIIGV    147
Phum_OR502AA_v1_g_05939   GRAMWHRTRWAIMYAWYFPLNYYN-----KSKYIYPHFWTSVVVWIDNPAIP-EPTIIGV    147
Phum_OR502AA_v1_g_18670   GRAIWHRKRWAIMYAWYFPGDFYF-----SDMLQFSHDWTSVVVWIDNPAIP-EPTIIGV    147
Phum_OR502AA_v1_g_19463   GRAVWHRDRWANMYTWYFPKNQAH-----DYEEGLRHDWANIVVWINNPEAP-VQKILGV    149
Phum_OR502AA_v1_g_08265   GRAIWHRDRWAIMYTWYFPKNQPG-----HYKVGLRHDWAHIVVWINNPDAS-VQTILGV    146
Phum_OR502AA_v1_g_05046   GRAIWHRDRWAIMYTWYFPKNQPK-----DDGVGHVHDWANIVVWINNPDAP-VQTILGV    148
Phum_OR502AA_v1_g_00914   GRAIWHRDRWAIMYTWYFPVNQPD--D--YYEYGHVHDWASLVVWINNPDAP-VQTILGV    149
Phum_OR502AA_v1_g_08901   GRAIWHRDRWAIMYTWYFPKNQPE-----YTKPGMQHDWAHIVVWINNPDAP-VQTILGV    148
Phum_OR502AA_v1_g_14668   GRAIWHRDRWAIMYTWYFPKNQPK-----DNGLGLRHDWAHIVVWINNPDAP-VQTILGV    148
Phum_OR502AA_v1_g_02635   SRSNWYKGKWAIMYAWYFPRGIQLS---YDYWYAHRHYWLYGIVWTDTPNPD-NSTILGV    148
Phum_OR502AA_v1_g_07619   SRSNWYKGKWAIMYAWYFPRGIQLS---YDYWYAHRHYWLYGIVWTDTPNPD-NSTILGV    148
Phum_OR502AA_v1_g_07620   SRSNWYKGKWAIMYAWYFPRGIQLS---YDYWYAHRHYWLYGIVWTDTPNPD-NSTILGV    148
Phum_OR502AA_v1_g_19643   SRSNWYKGKWAIMYAWYFPRGIQLS---YDYWYAHRHYWLYGIVWTDTPNPD-NSTILGV    148
Phum_OR502AA_v1_g_11522   ARSCLHKDKWATMYTWYLPKGKQP----DDFGIGHRHSWLYVVVWTDSPNPD-HSVILGV    148
Phum_OR502AA_v1_g_18234   GRADKVGSNWGTMYAWGFPTLNGT-------------VFYDVVVWINDPTPSDNQRIVSV    154
Phum_OR502AA_v1_g_08027   GRSGWVGTNWGTLYSYFIPYLGAG-------------NFMSVVVWINDPSPSSNQRVVAV    153
Phum_OR502AA_v1_g_13035   GRSGWVGTNWGTLYSYFIPYSGAG-------------IFMNVVVWINDPSPSSNQRIVAI    153
Phum_OR502AA_v1_g_02174   GRAIWHNQRWAII----------------------------IVVWIDNPAVP-EPTIIGV    124
```

Supplementary Figure S2. Necrosis and ethylene inducing peptide 1 (nep1)-like proteins (NLPs) in *Pseudoperonospora humuli*. *P. humuli* NLPs (green) are aligned with the *Phytophthora infestans* NPP1 (blue). Conserved cysteine residues and the domain important for necrosis are in black boxes

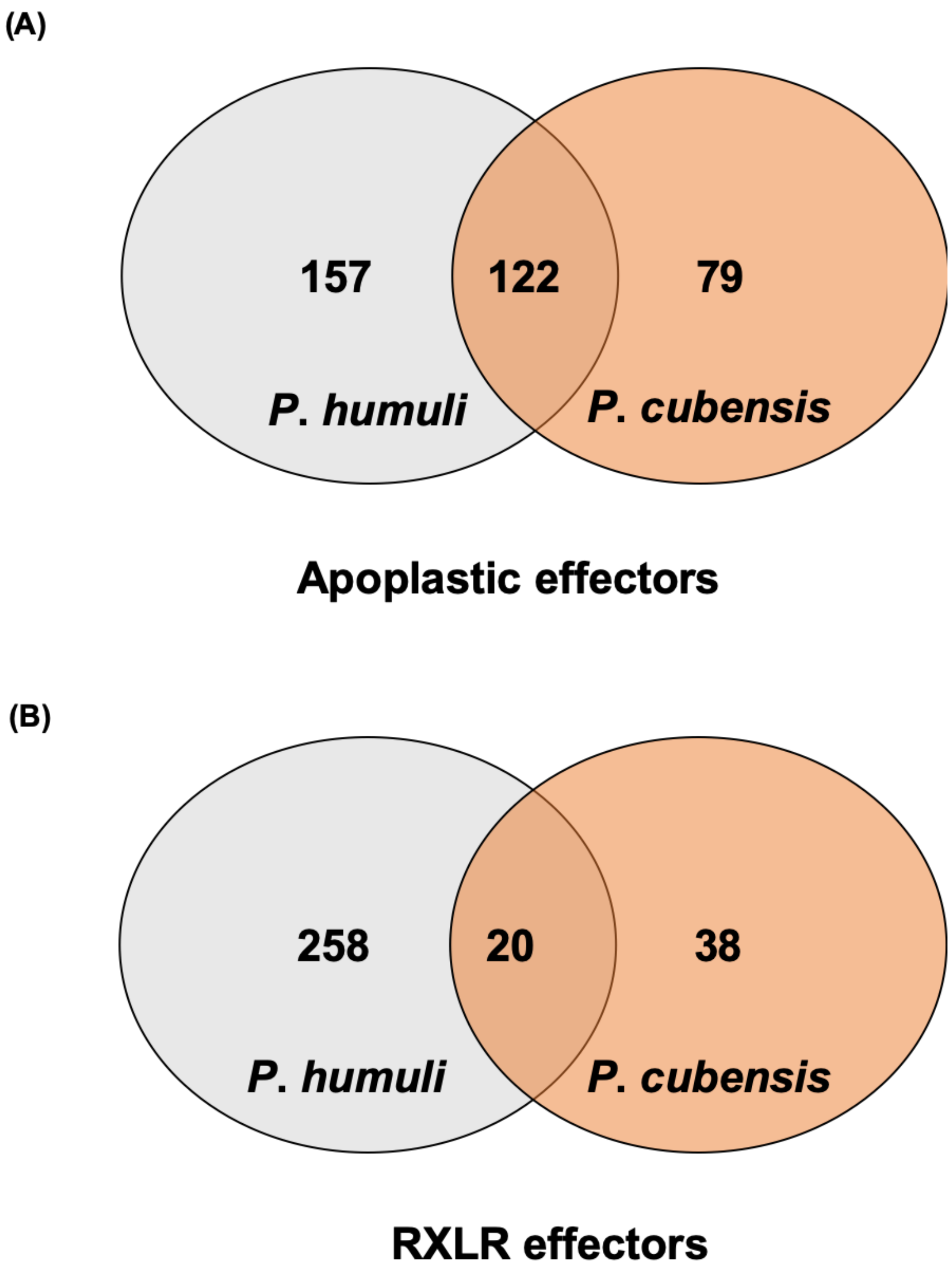

Supplementary Figure S3. Orthologous clusters of effectors in *Pseudoperonospora humuli* (grey) and *P. cubensis* (orange). (A) Apoplastic effectors; (B) RXLR effectors. Orthology analysis was performed using OrthoFinder v2.3.3.

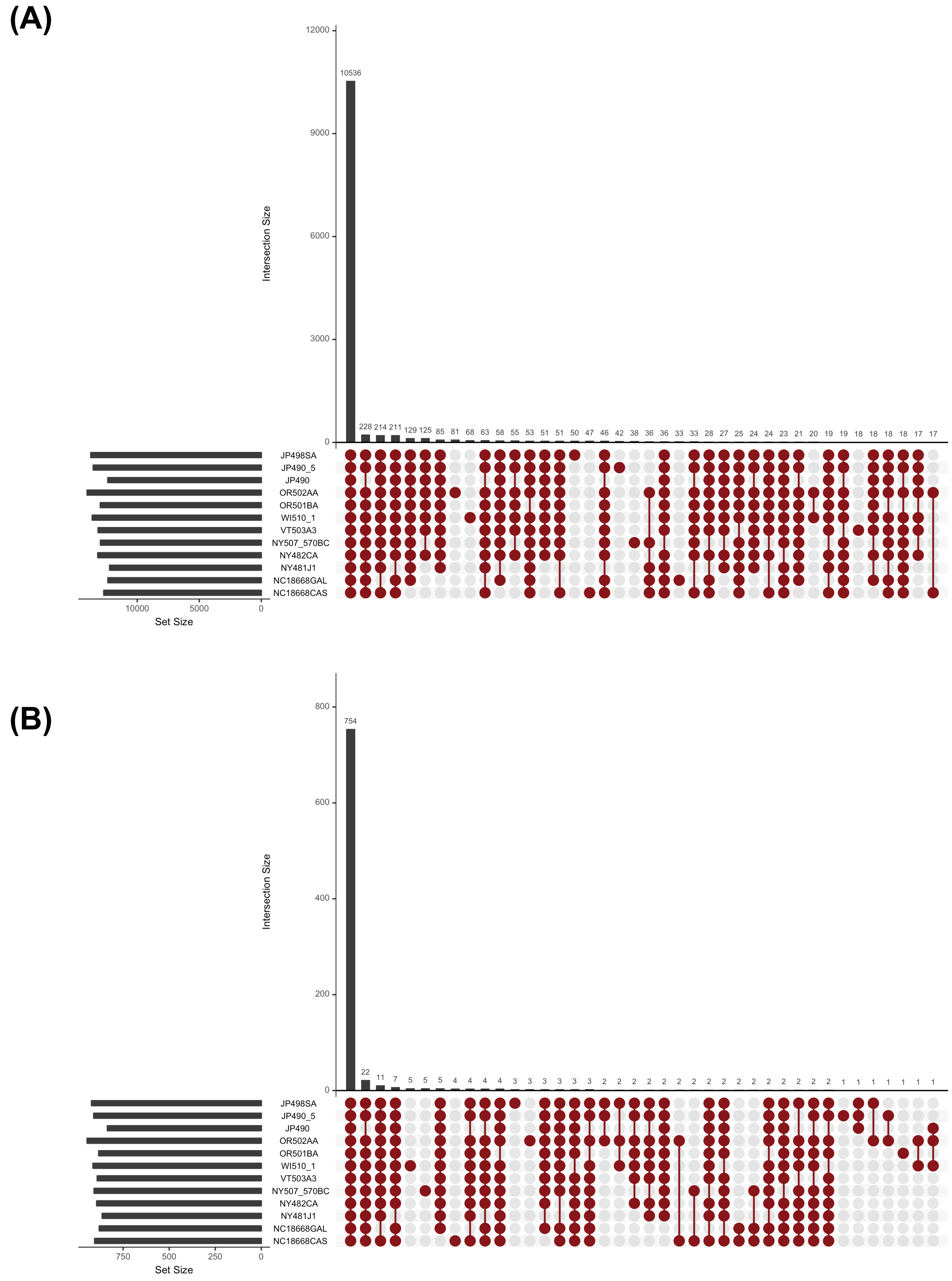

Supplementary Figure S4. Upset plots depicting the number of genes showing transcript evidence (RPKM >0) in the (A) total proteome, and (B) secretome of *Pseudoperonospora humuli*.

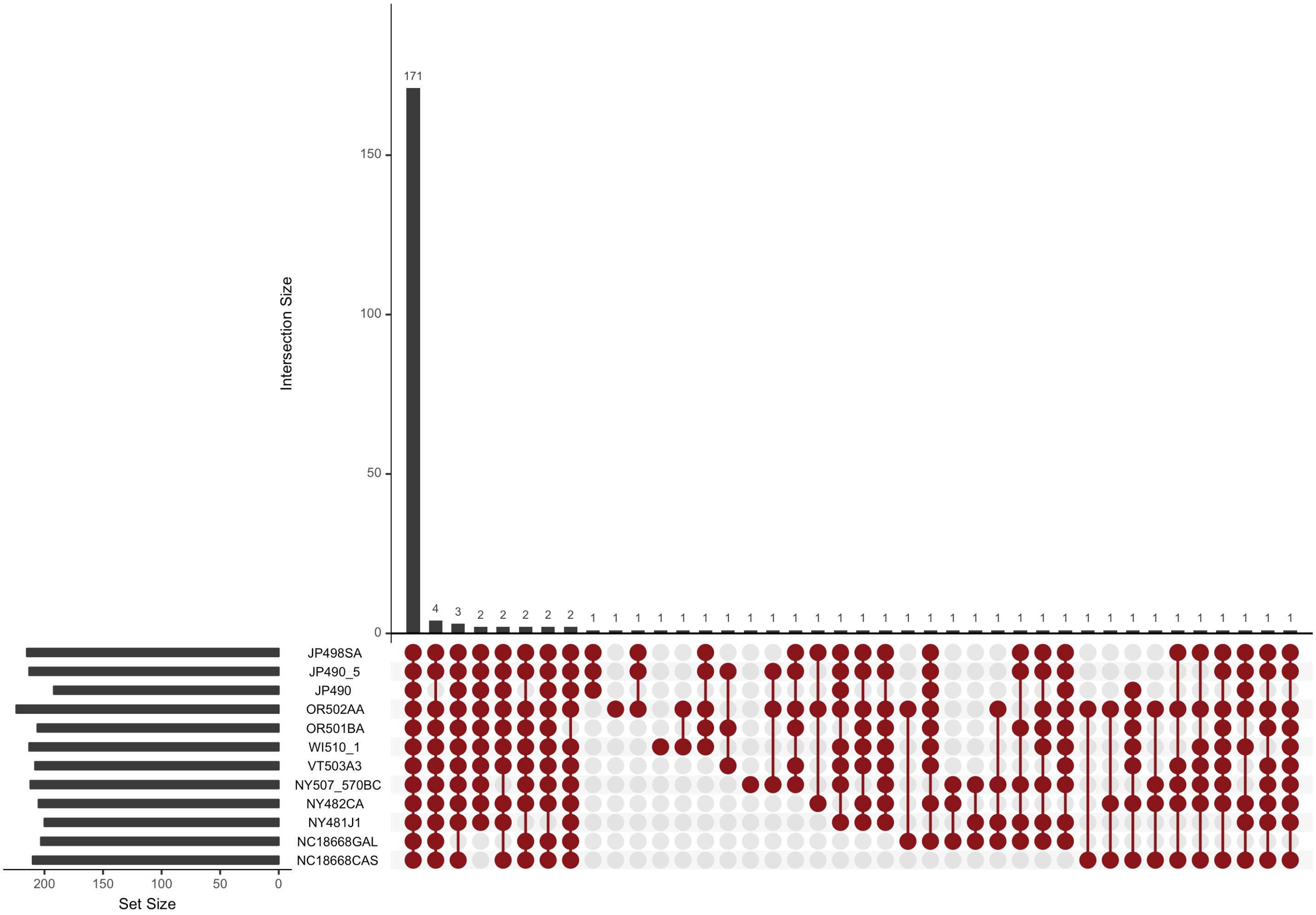

Fig. S5. Upset plot depicting apoplastic effectors that showed transcript evidence (RPKM >0) in 12 tested *Pseudoperonospora humuli* isolates.

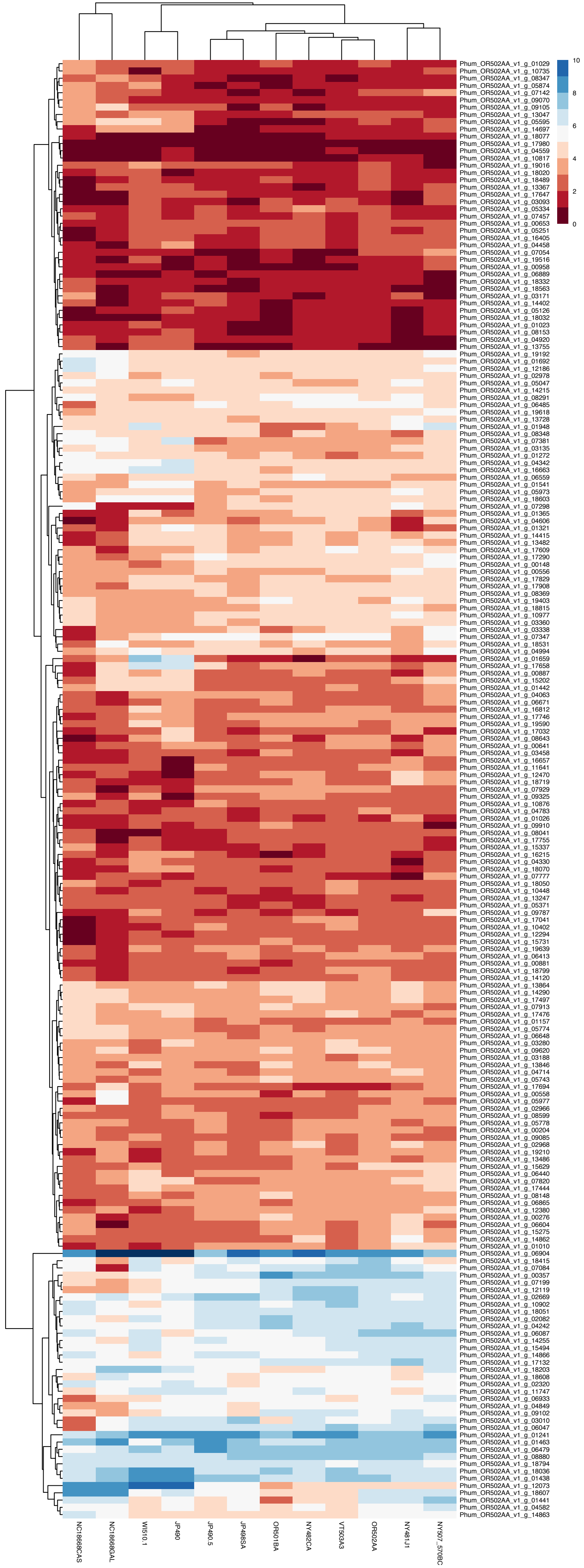

Phum_OR502AA_v1_g_01029
Phum_OR502AA_v1_g_10735
Phum_OR502AA_v1_g_08347
Phum_OR502AA_v1_g_05874
Phum_OR502AA_v1_g_07142
Phum_OR502AA_v1_g_09070
Phum_OR502AA_v1_g_09105
Phum_OR502AA_v1_g_13047
Phum_OR502AA_v1_g_05595
Phum_OR502AA_v1_g_14697
Phum_OR502AA_v1_g_18077
Phum_OR502AA_v1_g_17980
Phum_OR502AA_v1_g_04559
Phum_OR502AA_v1_g_10817
Phum_OR502AA_v1_g_19016
Phum_OR502AA_v1_g_18020
Phum_OR502AA_v1_g_18489
Phum_OR502AA_v1_g_13367
Phum_OR502AA_v1_g_17647
Phum_OR502AA_v1_g_03093
Phum_OR502AA_v1_g_05334
Phum_OR502AA_v1_g_07457
Phum_OR502AA_v1_g_00653
Phum_OR502AA_v1_g_05251
Phum_OR502AA_v1_g_16405
Phum_OR502AA_v1_g_04458
Phum_OR502AA_v1_g_07054
Phum_OR502AA_v1_g_19516
Phum_OR502AA_v1_g_00958
Phum_OR502AA_v1_g_06889
Phum_OR502AA_v1_g_18332
Phum_OR502AA_v1_g_18563
Phum_OR502AA_v1_g_03171
Phum_OR502AA_v1_g_14402
Phum_OR502AA_v1_g_05126
Phum_OR502AA_v1_g_18032
Phum_OR502AA_v1_g_01023
Phum_OR502AA_v1_g_08153
Phum_OR502AA_v1_g_04920
Phum_OR502AA_v1_g_13755
Phum_OR502AA_v1_g_19192
Phum_OR502AA_v1_g_01692
Phum_OR502AA_v1_g_12186
Phum_OR502AA_v1_g_02978
Phum_OR502AA_v1_g_05047
Phum_OR502AA_v1_g_14215
Phum_OR502AA_v1_g_08291
Phum_OR502AA_v1_g_06485
Phum_OR502AA_v1_g_19618
Phum_OR502AA_v1_g_13728
Phum_OR502AA_v1_g_01948
Phum_OR502AA_v1_g_08348
Phum_OR502AA_v1_g_07381
Phum_OR502AA_v1_g_03135
Phum_OR502AA_v1_g_01272
Phum_OR502AA_v1_g_04342
Phum_OR502AA_v1_g_16663
Phum_OR502AA_v1_g_06559
Phum_OR502AA_v1_g_01541
Phum_OR502AA_v1_g_05973
Phum_OR502AA_v1_g_18603
Phum_OR502AA_v1_g_07298
Phum_OR502AA_v1_g_01365
Phum_OR502AA_v1_g_04606
Phum_OR502AA_v1_g_01321
Phum_OR502AA_v1_g_14415
Phum_OR502AA_v1_g_13482
Phum_OR502AA_v1_g_17609
Phum_OR502AA_v1_g_17290
Phum_OR502AA_v1_g_00148
Phum_OR502AA_v1_g_00556
Phum_OR502AA_v1_g_17829
Phum_OR502AA_v1_g_17908
Phum_OR502AA_v1_g_08369
Phum_OR502AA_v1_g_19403
Phum_OR502AA_v1_g_18815
Phum_OR502AA_v1_g_10977
Phum_OR502AA_v1_g_03360
Phum_OR502AA_v1_g_03338
Phum_OR502AA_v1_g_07347
Phum_OR502AA_v1_g_18531
Phum_OR502AA_v1_g_04994
Phum_OR502AA_v1_g_01659
Phum_OR502AA_v1_g_17658
Phum_OR502AA_v1_g_00887
Phum_OR502AA_v1_g_15202
Phum_OR502AA_v1_g_01442
Phum_OR502AA_v1_g_04063
Phum_OR502AA_v1_g_06671
Phum_OR502AA_v1_g_16812
Phum_OR502AA_v1_g_17746
Phum_OR502AA_v1_g_19590
Phum_OR502AA_v1_g_17032
Phum_OR502AA_v1_g_08643
Phum_OR502AA_v1_g_00641
Phum_OR502AA_v1_g_03458
Phum_OR502AA_v1_g_16657
Phum_OR502AA_v1_g_11641
Phum_OR502AA_v1_g_12470
Phum_OR502AA_v1_g_18719
Phum_OR502AA_v1_g_07929
Phum_OR502AA_v1_g_09325
Phum_OR502AA_v1_g_10876
Phum_OR502AA_v1_g_04783
Phum_OR502AA_v1_g_01026
Phum_OR502AA_v1_g_09910
Phum_OR502AA_v1_g_08041
Phum_OR502AA_v1_g_17755
Phum_OR502AA_v1_g_15337
Phum_OR502AA_v1_g_16215
Phum_OR502AA_v1_g_04330
Phum_OR502AA_v1_g_18070
Phum_OR502AA_v1_g_07777
Phum_OR502AA_v1_g_18050
Phum_OR502AA_v1_g_10448
Phum_OR502AA_v1_g_13247
Phum_OR502AA_v1_g_05371
Phum_OR502AA_v1_g_09787
Phum_OR502AA_v1_g_17041
Phum_OR502AA_v1_g_10402
Phum_OR502AA_v1_g_12294
Phum_OR502AA_v1_g_15731
Phum_OR502AA_v1_g_19639
Phum_OR502AA_v1_g_06413
Phum_OR502AA_v1_g_00881
Phum_OR502AA_v1_g_18799
Phum_OR502AA_v1_g_14120
Phum_OR502AA_v1_g_13864
Phum_OR502AA_v1_g_14290
Phum_OR502AA_v1_g_17497
Phum_OR502AA_v1_g_07913
Phum_OR502AA_v1_g_17476
Phum_OR502AA_v1_g_01157
Phum_OR502AA_v1_g_05774
Phum_OR502AA_v1_g_06648
Phum_OR502AA_v1_g_03280
Phum_OR502AA_v1_g_09620
Phum_OR502AA_v1_g_03188
Phum_OR502AA_v1_g_13846
Phum_OR502AA_v1_g_04714
Phum_OR502AA_v1_g_05743
Phum_OR502AA_v1_g_17694
Phum_OR502AA_v1_g_00558
Phum_OR502AA_v1_g_05977
Phum_OR502AA_v1_g_02966
Phum_OR502AA_v1_g_08599
Phum_OR502AA_v1_g_05778
Phum_OR502AA_v1_g_00204
Phum_OR502AA_v1_g_09085
Phum_OR502AA_v1_g_02968
Phum_OR502AA_v1_g_19210
Phum_OR502AA_v1_g_13486
Phum_OR502AA_v1_g_15629
Phum_OR502AA_v1_g_06440
Phum_OR502AA_v1_g_07820
Phum_OR502AA_v1_g_17444
Phum_OR502AA_v1_g_08148
Phum_OR502AA_v1_g_06865
Phum_OR502AA_v1_g_12380
Phum_OR502AA_v1_g_00276
Phum_OR502AA_v1_g_06604
Phum_OR502AA_v1_g_14862
Phum_OR502AA_v1_g_15275
Phum_OR502AA_v1_g_01010
Phum_OR502AA_v1_g_06904
Phum_OR502AA_v1_g_18415
Phum_OR502AA_v1_g_07084
Phum_OR502AA_v1_g_00357
Phum_OR502AA_v1_g_07199
Phum_OR502AA_v1_g_12119
Phum_OR502AA_v1_g_02669
Phum_OR502AA_v1_g_10902
Phum_OR502AA_v1_g_18051
Phum_OR502AA_v1_g_02082
Phum_OR502AA_v1_g_04242
Phum_OR502AA_v1_g_06087
Phum_OR502AA_v1_g_14255
Phum_OR502AA_v1_g_15494
Phum_OR502AA_v1_g_14866
Phum_OR502AA_v1_g_17132
Phum_OR502AA_v1_g_18203
Phum_OR502AA_v1_g_18608
Phum_OR502AA_v1_g_02320
Phum_OR502AA_v1_g_11747
Phum_OR502AA_v1_g_06933
Phum_OR502AA_v1_g_04849
Phum_OR502AA_v1_g_09102
Phum_OR502AA_v1_g_03010
Phum_OR502AA_v1_g_06047
Phum_OR502AA_v1_g_01241
Phum_OR502AA_v1_g_01463
Phum_OR502AA_v1_g_06479
Phum_OR502AA_v1_g_08880
Phum_OR502AA_v1_g_18794
Phum_OR502AA_v1_g_18036
Phum_OR502AA_v1_g_01438
Phum_OR502AA_v1_g_12073
Phum_OR502AA_v1_g_18607
Phum_OR502AA_v1_g_01441
Phum_OR502AA_v1_g_04582
Phum_OR502AA_v1_g_14863
NC18668CAS
NC18666GAL
WI510.1
JP490
JP490.5
JP498SA
OR501BA
NY482CA
VT503A3
OR502AA
NY481J1
NY507_570BC
10
8
6
4
2
0

Supplementary figure S6. RNAseq expression of RXLRs in the sporangia of *Psuedoperonospora humuli* isolates. Reads per kilobase million (RPKM) values were log_2 transformed and used to generate heatmap.

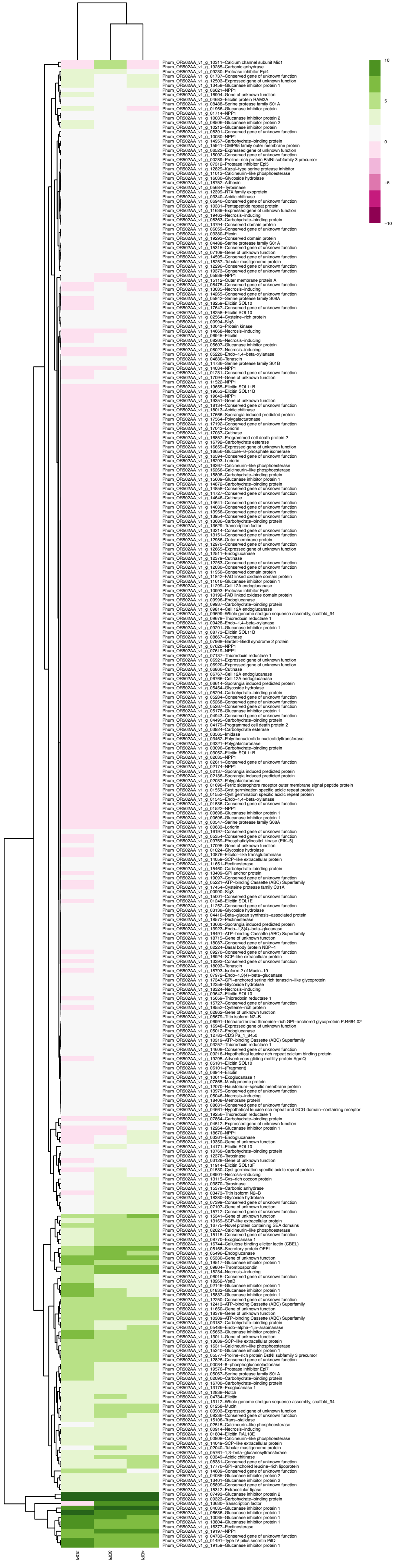

10
5
0
-5
-10
2DPI
3DPI
4DPI

Supplementary figure S7. RNAseq expression of apoplastic effectors of *Psuedoperonospora humuli* during infection. Reads per kilobase million (RPKM) values were log_2 transformed and used to generate heatmap.

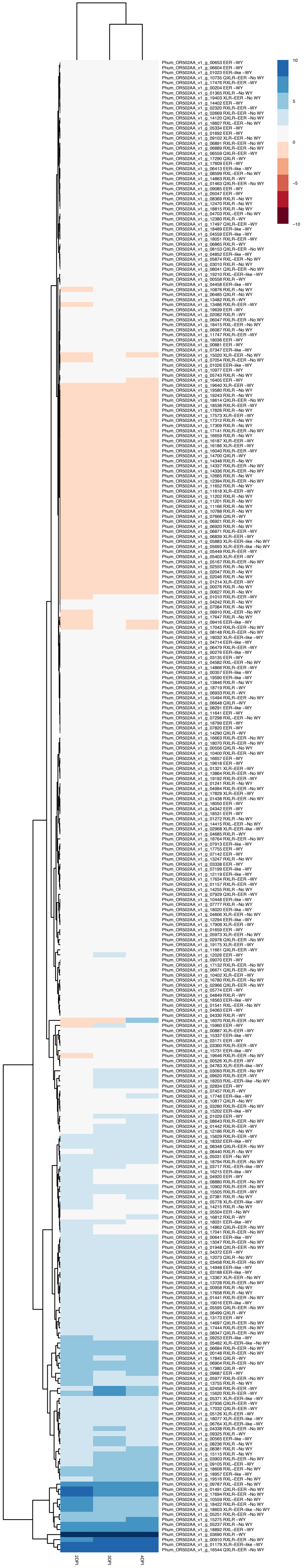
10
5
0
−5
−10

Supplemetary Figure S8. Heatmap showing the expression pattern of RXLR effectors of *Pseudoperonospora humuli* (OR502AA) during infection.

RPKM values obtained at different time-points were log2 transformed with respect to the RPKM values of the sporangia and used to generate heatmap. (DPI: days post infection).
